# Choice of outcome measure methods affects reported results in stapes surgery. Data from the Swedish quality register for otosclerosis surgery

**DOI:** 10.1007/s00405-025-09567-7

**Published:** 2025-08-13

**Authors:** Ulrica Thunberg, Erika Elfstrand, Lars Lundman

**Affiliations:** 1 Department of Otorhinolaryngology, Örebro University Hospital, 70185 Örebro, SE Sweden; 2 Faculty of Medicine and Health, Örebro University, Örebro, Sweden; 3 Department of Otorhinolaryngology, Central Hospital, Karlstad, Sweden

**Keywords:** Otosclerosis, Surgery, Outcome measure, Audiometry

## Abstract

**Purpose:**

To investigate differences in stapes surgery outcome when using different calculation methods.

**Methods:**

Audiometric data were retrieved from the Swedish Quality Register for Otosclerosis Surgery for 3159 surgeries conducted during 2013–2024 with complete pre- and postoperative pure tone audiometry measurements of 0.5, 1, 2, 3, and 4 kHz. Outcomes were calculated in two ways: using 3 kHz versus 4 kHz in four-frequency averaging of air conduction, bone conduction, air–bone gap, and gain.

**Results:**

Postoperative air–bone gap improved from 10.0 dB to 7.6 dB when using 3 kHz instead of 4 kHz, and the proportion of successful cases (defined as a postoperative air–bone gap ≤ 10 dB) increased from 62.9% to 79.0%. When 3 kHz was replaced by averaging 2 and 4 kHz, a significantly larger four-frequency air–bone gap was seen in comparison to using the measured 3 kHz.

**Conclusion:**

This study showed a significant impact on postoperative four-frequency air–bone gap depending on whether the frequency 3 kHz or 4 kHz was included in averaging. The effect appears to be larger than previously described. Bone conduction measurements at 4 kHz produced an anomalous and too low measured hearing level, resulting in a disproportionate impact of the 4 kHz frequency on postoperative four-frequency air–bone gap. Comparison of study results that use different calculation methods should therefore be done with caution. We advocate using the methods recommended by the Committee on Hearing and Equilibrium of the American Academy of Otolaryngology–Head and Neck Surgery for uniformity in presentation of results.

## Introduction

Otosclerosis is a disease that causes stiffness of the stapes footplate. This mechanical blocking of sound waves can be surgically treated by a continuous prosthesis into the inner ear. There is a considerable difference in how hearing results after stapes surgery are analysed and presented, which affects the interpretation of results. Averaging of audiometric data into numbers for description of results is standard for benchmarking and comparison of results; but methods vary, and so it is of great value to evaluate the impact of different choices as a basis for outcome measures.

The air–bone gap (ABG) measurement together with air conduction (AC) and bone conduction (BC) has a crucial role in determining the degree of hearing loss as conductive, sensorineural, or mixed. Audiometry is usually performed including frequencies of 0.25–8 kHz for AC and 0.5–4 kHz for BC. The ABG is the AC threshold minus the BC threshold at a certain hearing frequency, and can be 0 dB, positive, or negative. Patients with pathology in the middle ear have in most cases a conductive hearing loss, and often, when otosclerosis is present, also a simultaneous depression of BC thresholds.

Margolis et al. showed that high frequency BC hearing above 3 kHz was elevated both in normal hearers and in patients with sensorineural hearing loss, leading to a “false” ABG that increases with increased hearing loss [1]. The 1995 guidelines from the Committee on Hearing and Equilibrium of the American Academy of Otolaryngology–Head and Neck Surgery (AAO-HNS) recommend that results after middle ear surgery should be classified as follows: when bins are used for ABG, they should be constructed as 0–10 dB, 11–20 dB, 21–30 dB, and > 30 dB, and when audiogram data are averaged into ABG, the frequencies 0.5, 1, 2, and 3 kHz should be used from audiograms performed at the same time [2, 3]. However, this has not become a uniform standard when presenting hearing results after stapes surgery [4].

A study by Berliner et al. from 1996 included data from 240 stapes surgeries. The authors found a slightly lower success rate when 4 kHz was included in pure tone average for the four frequencies rather than 3 kHz, and consequently a greater impact on the average postoperative ABG [5]. De Bruijn et al. elaborated the consequences of using different AC and BC frequencies for presenting results in otosclerosis surgery, and concluded that using four-frequency calculations including 3 or 4 kHz is recommended [6]. In many countries 3 kHz is not included in standard audiometry, and so it is common to substitute 3 kHz with the mean values for 2 and 4 kHz, which is suggested as a good enough substitution [7]. However, in Sweden, 3 kHz is measured as standard when performing audiometry.

The objective of this study was to utilize audiometric data from the Swedish Quality Register for Otosclerosis Surgery (SQOS) to describe and evaluate the differences between four-frequency results presented with 3 kHz or 4 kHz. A second objective was to examine the impact on average ABG.

## Materials and methods

The SQOS was established in 2004 [8], and from 2013 onwards has included more than 90% of the stapes surgeries performed in Sweden in patients older than 15 years. This proportion is known, as all surgeries in Sweden are registered in the National Patient Register administered by the National Board of Health and Welfare. Twenty-four surgical units, including all eight university clinics, have entered data into the register. Moreover, 90% of the surgeries entered into the register from 2013 to 2022 have complete postoperative audiometric data, which means that approximately 81% of all stapes surgeries performed in Sweden during this period have follow-up data.

The results of the pre- and postoperative pure-tone audiometry are entered manually by the user into the database. Pure tone audiograms are complete, since entry of each frequency value is mandatory, and the structure of the register form minimizes entry of inaccurate values. When hearing thresholds for individual frequencies are undetectable due to profound hearing loss, AC is set to 130 dB hearing level and BC to 75 dB hearing level. A one-year follow-up is recommended, but follow-up data can be entered at any time after surgery. The database is validated yearly, and is corrected according to original audiograms if necessary. The validation procedures and results are presented on the SQOS website [9]. For the present study, all registered surgeries with postoperative audiometric data from 2013 to 2024 were retrieved for analysis on 1 January 2025. In Sweden, all clinical measurement of hearing is performed by licensed audiologists who have undergone a three-year undergraduate program. The methodology used for measurement is stable over years, and is in line with the Swedish TeMA Metod guide [10] as well as guidelines from the American Speech–Language–Hearing Association. As far as we know, when it comes to registering data in the SQOS, all hearing units in Sweden work according to these standards for pure tone measurement of hearing.

### Audiometry

Hearing levels are registered in the SQOS at 0.25, 0.5, 1, 2, 3, 4, 6, and 8 kHz for AC and at 0.5, 1, 2, 3 and 4 kHz for BC. Pre- and postoperative ABG were calculated for each frequency between 0.5 and 4 kHz. Mean pre- and postoperative BC and AC pure tone average (PTA4) and ABG were calculated for 0.5, 1, 2, and 3 kHz and for 0.5, 1, 2, and 4 kHz respectively. Postoperative BC and AC were used for postoperative ABG calculations. The results were tabulated, and illustrated in audiometry figures and in Amsterdam hearing evaluation plots (AHEPs) [11].

### Statistics

The Shapiro–Wilk test was performed to confirm the normality of the audiometric data. Comparisons of continuous audiometric data including means of AC, BC, ABG, and hearing gain were conducted using the t-test. Descriptive statistics included calculation of mean and standard deviation. Non-parametric data were compared with the Wilcoxon signed- rank test. A two-sided p-value < 0.05 was considered statistically significant. The analyses were performed using version 29 of IBM SPSS Statistics (IBM Corp., Armonk, NY, USA).

## Results

### Subjects

A total of 3186 surgical procedures were identified with complete pre- and postoperative audiograms. Of these, 20 were aborted operations and 7 had audiograms with apparently inaccurate values such as confusion of operated side or confusion of BC and AC; all 27 were excluded.

The remaining 3159 surgical procedures were performed in 2762 patients and consisted of 2075 (65.7%) primary operations in patients with no previous stapes surgery, 829 (26.2%) primary operations on the second ear, and 255 (8.1%) revision cases. Mean and median age at the time of surgery were both 50 years, and 62.0% of the surgeries were performed on female patients. Small fenestra stapedotomy using various lasers and microdrill techniques was the dominant surgical procedure (98.7%) in the primary surgery cases, and the most common prosthesis was a platinum-Teflon piston prosthesis (83.1%). A detailed description of surgical techniques is not relevant for this study.

### Hearing

**Fig. 1:**
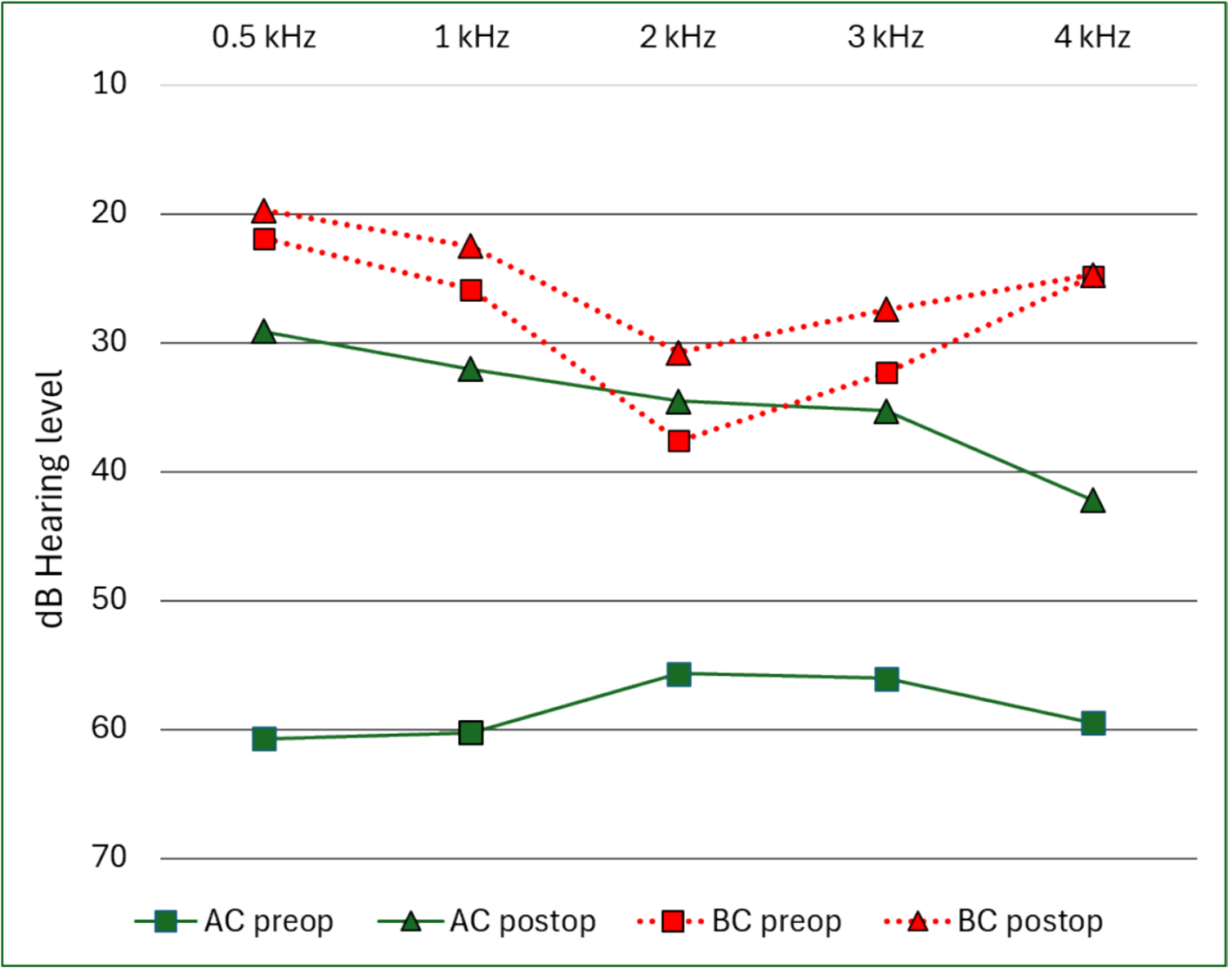
Mean pre- and postoperative hearing thresholds for 0.5, 1, 2, 3, and 4 kHz air conduction (AC) and bone conduction (BC) from 3159 otosclerosis surgeries

Results are presented in terms of the hearing frequencies included for pre- and postoperative AC and BC thresholds when averaging hearing results and computing ABG. The differences between calculations including 3 kHz or 4 kHz for preoperative and postoperative AC and BC PTA4 and mean ABG, as well as gain for these calculations, were all statistically significant (Fig. 1, Table 1 and 2).

**Table 1:** Mean pre-and postoperative air- and bone conduction thresholds, air-bone gaps, and gains for each frequency between 0.5 and 4 kHz in 3159 cases operated for otosclerosis

Measure (kHz)	Preop (dB)	SD	Postop (dB)	SD	Gain (dB)	SD
AC 0.5	60.7	16.7	29.1	17.2	31.6	15.9
AC 1	60.2	18.3	32.0	18.3	28.2	15.1
AC 2	55.7	20.8	34.5	20.1	21.1	14.8
AC 3	56.0	23.0	35.3	22.0	20.7	15.6
AC 3(^2/4^)	57.6	22.0	38.4	20.9	19.2	14,2
AC 4	59.5	25.4	42.2	23.9	17.2	16.5
BC 0.5	21.9	14.2	19.8	15.4	2.2	10.2
BC 1	25.9	15.1	22.5	15.9	3.4	10.2
BC 2	37.6	16.5	30.7	18.0	6.9	9.9
BC 3	32.3	18.0	27.4	19.5	4.9	9.5
BC 3(^2/4^)	31.2	17.1	27.7	18.3	3,5	8,2
BC 4	24.8	19.8	24.7	20.9	0.1	10.3
ABG 0.5	38.8	13.1	9.3	10.4	29.4	15.3
ABG 1	34.4	12.2	9.5	8.9	24.8	14.1
ABG 2	18.1	12.0	3.8	8.3	14.3	13.6
ABG 3	23.7	12.3	7.9	8.5	15.8	13.5
ABG (^2/4^)	26.4	11.2	10.7	8.1	15.7	12.6
ABG 4	34.7	14.2	17.5	11.3	17.2	15.8

Fotnote: AC = air conduction, BC = bone conduction, ABG = air bone gap, SD = standard deviation, (^2/4^) = average of 2 and 4 kHz

**Table 2:** Mean four frequency pre-and postoperative air- and bone conduction thresholds and air-bone gaps including 3 kHz or 4 kHz in 3159 patients operated for otosclerosis

Preoperative			Postoperative		
Air conduction dB HL (SD)		Sign	Air conduction dB HL (SD)		Sign
0.5, 1, 2, 3	0.5, 1, 2, 4		0.5, 1, 2, 3	0.5, 1, 2, 4	
58.2 (18.1)	59.0 (18.4)	< 0.001	32.7 (17.9)	34.5 (18.0)	< 0.001
Bone conduction dB HL (SD)			Bone conduction dB HL(SD)		
0.5, 1, 2, 3	0.5, 1, 2, 4		0.5, 1, 2, 3	0.5, 1, 2, 4	
29.4 (14.2)	27.5 (14.3)	< 0.001	25.1 (15.3)	24.4 (15.4)	< 0.001
Air–bone gap dB (SD)			Air–bone gap dB (SD)		
0.5,1,2,3	0.5,1,2,4		0.5,1,2,3	0.5,1,2,4	
28.7 (9.9)	31.5 (10.1)	< 0.001	7.6 (7.0)	10.0 (7.3)	< 0.001

Fotnote: *dB* decibel, *HL* hearing level, *SD* standard deviation. Significance with pairwise t-test

### Postoperative air–bone gap and success rate

**Fig. 2:**
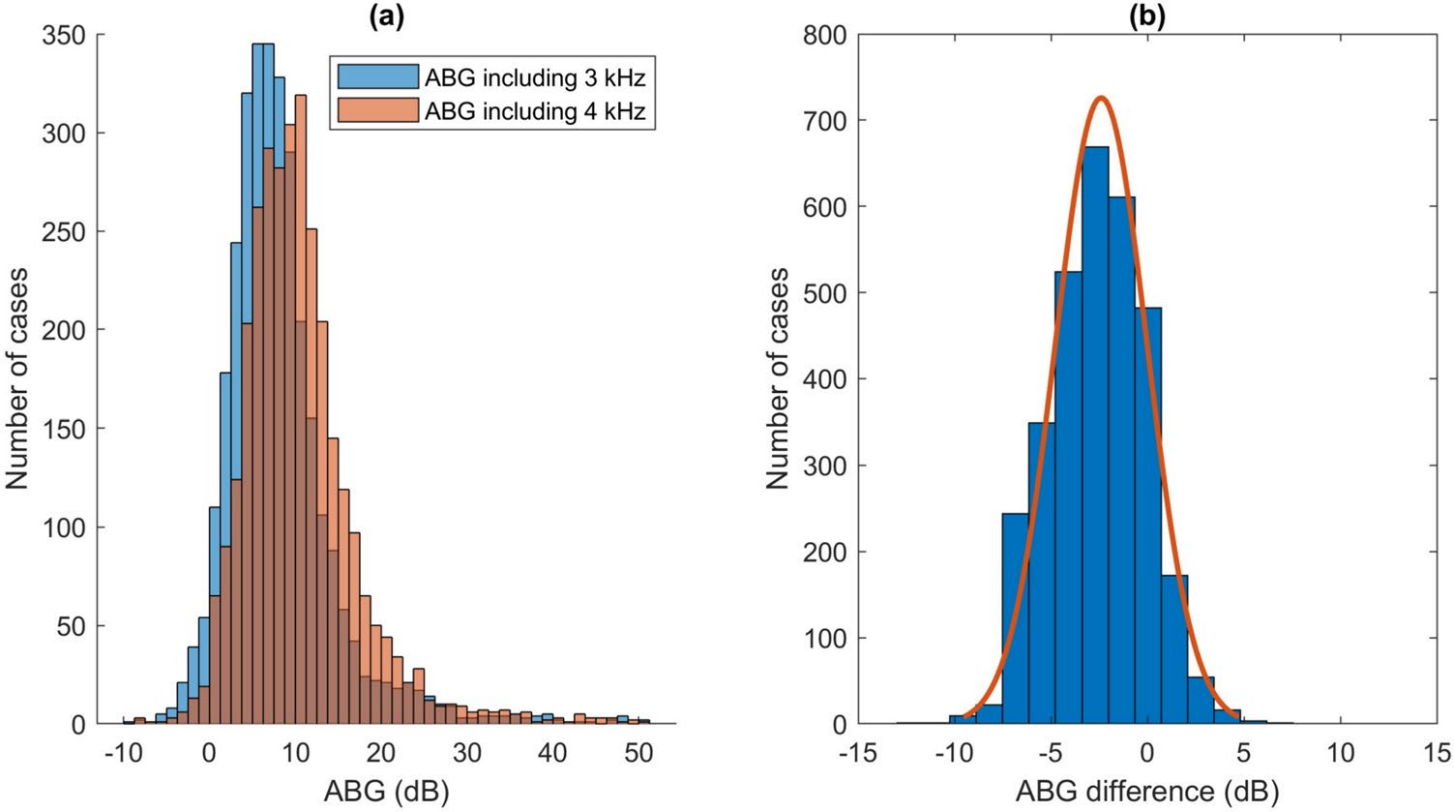
a) Distribution of postoperative mean four-frequency air–bone gap (ABG) using 3 or 4 kilohertz (kHz) b) Distribution of the individual difference using 3 and 4 kHz for calculation of four-frequency postoperative ABG

**Fig. 3:**
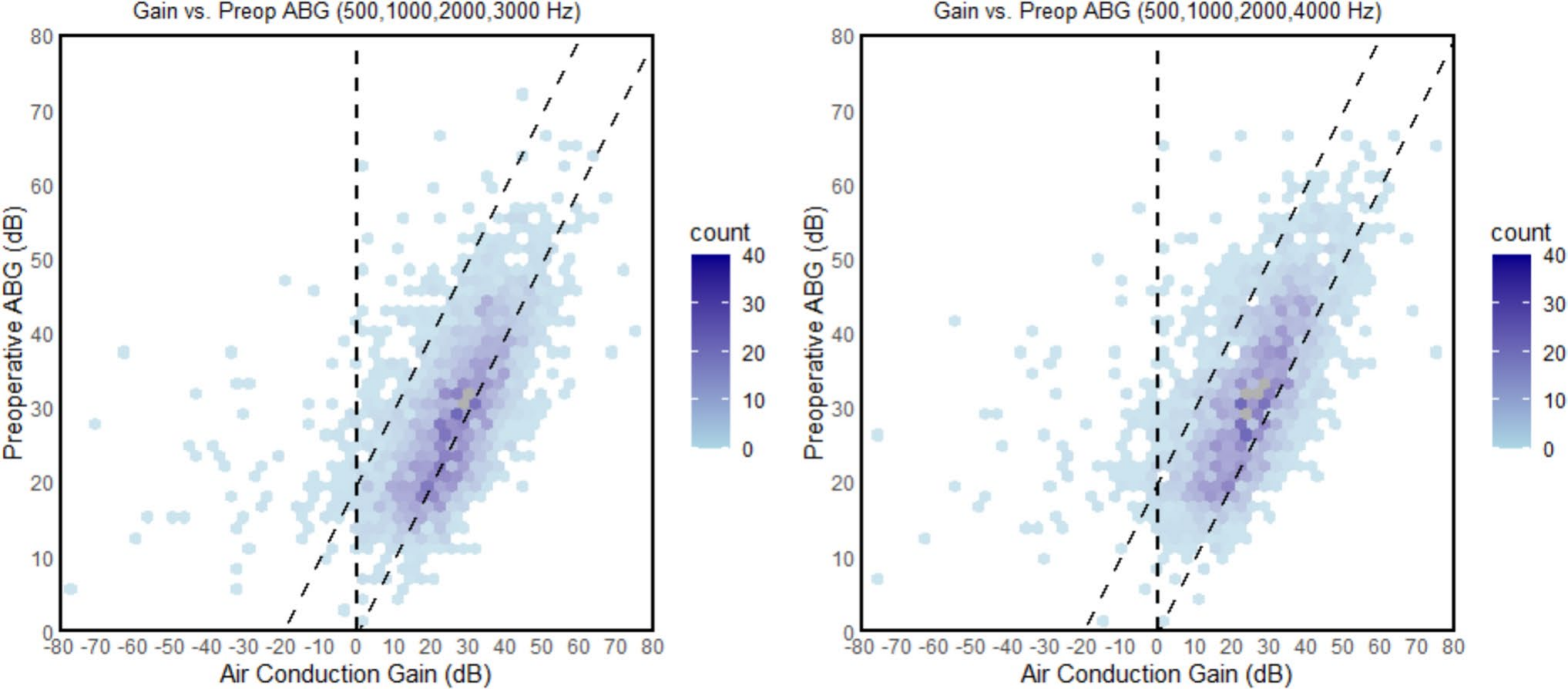
Modified Amsterdam hearing evaluation plots presenting the differences for preoperative four-frequency air–bone gap (ABG) versus air conduction gain using 3 or 4 kilohertz (kHz) in the averaging calculation. Since many data points are stacked upon each other, different intensities of colour are used to illustrate the bulk of the data points in a semi-three-dimensional fashion

Mean postoperative four-frequency ABG improved from 10.0 dB to 7.6 dB when using 3 kHz instead of 4 kHz. Mean postoperative ABG was 17.5 dB at 4 kHz and 7.9 dB at 3 kHz, and consequently 4 kHz accounted for a larger proportion of four-frequency ABG in comparison to 3 kHz (Fig. 1, Table 2 and 3). Figure 2 illustrates (a) the distribution of mean postoperative four-frequency ABG depending on whether 3 kHz or 4 kHz was used for the calculation, and (b) the distribution of the difference. The modified AHEP plot also showed a clear shift toward the area on the right side of the lower oblique line when 4 kHz was replaced by 3 kHz, indicating a higher proportion of “overclosure” (Fig. 3). It is worth noting that cases illustrated in the AHEP with preoperative mean ABG < 10 dB can be attributed to revision cases operated because of balance or dizziness problems rather than hearing loss.

**Table 3:** Postoperative four frequency air-bone gaps in bins when comparing four-frequency calculations using 3 kHz or 4 kHz

	Air–bone gap	
	0.5, 1, 2, 3 kHz (%)	0.5, 1, 2, 4 kHz (%)
≤ 10 dB	79.0	62.9
≤ 20 dB	95.3	93.8
≤ 30 dB	98.3	98.8

Footnote; *dB* decibel, *kHz* kilohertz, n = 3159 surgical procedures

Postoperative mean four-frequency ABG was 8.3 dB when 3 kHz measurements were replaced with the averages for 2 kHz and 4 kHz, which was significantly larger than the result of calculation with the measured values for 3 kHz (7.6 dB, p < 0.001). This difference was mainly caused by the low mean 4 kHz AC as demonstrated in Fig. 1.

The proportion of cases with closure of ABG to ≤ 10 dB increased from 62.9% to 79.0% when using 3 kHz instead of 4 kHz. Furthermore, the proportion with ABG ≤ 20 dB increased from 93.8% to 95.3% and the proportion with ≤ 30 dB decreased from 98.8% to 98.3% (Table 3).

### Postoperative air conduction

Postoperative PTA4 AC differed between the two methods (32.7 dB when using 3 kHz vs. 34.5 dB when using 4 kHz). The proportion of surgeries with four-frequency AC gain ≥ 20 dB was 72.9% when 3 kHz was used vs. 69.7% when 4 kHz was used. The difference was statistically significant (p < 0.001).

### Postoperative bone conduction

Postoperative PTA4 BC was 25.1 dB when 3 kHz was used and 24.4 dB when 4 kHz was used. Mean threshold shift for four-frequency BC was 4.3 dB when using 3 kHz and 3.1 dB when using 4 kHz. Mean BC threshold shift was largest for 2 kHz (6.9 dB). At 4 kHz there was virtually no threshold shift. Overclosure at 2 kHz was evident (Fig. 1, Table 1 and 2).

## Discussion

In this study we used SQOS register data obtained from 3159 stapes surgery cases to explore the impact of different choices of methods when averaging and presenting audiometric results. The main finding was that the proportion of cases with closure of four-frequency ABG to ≤ 10 dB increased from 62.9% to 79.0% when including 3 kHz instead of 4 kHz. Moreover, the mean postoperative four-frequency ABG improved by 2.4 dB from 10.0 dB to 7.6 dB.

Our findings are in accordance with a study by Kisilevsky et al. presenting audiometric results from 1145 primary stapedotomies, where an ABG of ≤ 10 dB was seen in 75.2% of patients when averaging 0.5, 1, 2, and 4 kHz and in 81.8% of patients when averaging 0.5, 1, 2, and 3 kHz. Furthermore, mean postoperative ABG improved by 1.5 dB from 7.6 dB to 6.1 dB when using 3 kHz instead of 4 kHz [12]. Similar results were found by Berliner et al. in a study including 240 surgeries, showing that postoperative mean ABG improved by 1.8 dB from 11.9 dB to 10.1 dB when 3 kHz was used instead of 4 kHz. This led to a success rate (ABG ≤ 10 dB) of 67.9% instead of 61.6% [5].

Since year 2000 there are only published a few other studies were from postoperative mean four-frequency ABG can be calculated using both methods (3 kHz and 4 kHz). For eight of these studies, inclusion of 3 kHz resulted in a smaller postoperative mean four-frequency ABG [8, 12–19] and in three, a larger ABG than using 4 kHz [20–22]. However, most of these studies were not based on as many cases as our results, which is a strength of the present study.

The degree of ABG closure is the most common outcome used when reporting comparisons of the results of stapes surgery. The AAO-HNS Committee on Hearing and Equilibrium stated in 1995 that data sets using 0.5, 1, and 2 kHz or 0.5, 1, 2, and 4 kHz can be compared directly with data sets using 0.5, 1, 2, and 3 kHz, but recommended that future prospective studies should standardise on using the average of 0.5, 1, 2, and 3 kHz [2]. Nevertheless, in a review of 51 stapes surgery articles published between 2005 and 2014 studying ABG closure calculated using the method outlined by the Committee on Hearing and Equilibrium (0.5, 1, 2, and 3 kHz), it was found that only 45.1% of studies actually used measured 3 kHz when calculating the four-frequency average; the remainder either averaged the values for 2 and 4 kHz, or substituted 4 kHz for 3 kHz [4].

In many institutions it is not standard to measure 3 kHz; this appears to be one reason for averaging or substituting 3 kHz in retrospective studies, which constitute the majority of published stapes surgery results from the past two decades. In the present study, calculation of postoperative four-frequency ABG by using the average of 2 kHz and 4 kHz resulted in a statistically significant larger ABG compared to using measured thresholds at 3 kHz. Gurgel et al. investigated 2170 audiograms and concluded that averaging 2 and 4 kHz AC can accurately approximate 3 kHz AC [7], but to our knowledge, no studies have investigated whether this substitution can be used when calculating postoperative ABG for middle ear surgery evaluation. In this study, the difference between calculation of 3 kHz using an average of 2 and 4 kHz or by measuring 3 kHz when calculating postoperative ABG was statistically significant but quite small (0.7 dB), and so its clinical relevance is questionable. It is therefore likely that calculating 3 kHz from 2 and 4 kHz can be done without greatly hampering the results.

According to Margolis et al., the BC frequency 4 kHz is unstable, and may cause an “anomalous” ABG at 4 kHz both in normal ears and in ears with sensorineural hearing loss. The ABG at the specific frequency of 4 kHz increases with increased hearing loss, and can be as much as 20 dB in ears with AC hearing threshold worse than 60 dB. The phenomenon can also be traced in lower frequencies, but to a lesser degree. On the basis of their findings, Margolis et al. suggested that 4 kHz BC should be corrected in standard audiometry [23]. However, it remains unclear whether this phenomenon occurs and has an important impact on calculating pre- and postoperative mean ABG in patients with conductive hearing loss or mixed hearing loss. In light of the results of Margolis et al., it is possible that the relatively low value for mean 4 kHz BC in this study is anomalous, and therefore incorrectly impairs the postoperative mean four-frequency ABG when 4 kHz is included instead of 3 kHz.

The present study included a very large population, which led to statistically significant results in most comparisons. The numerical differences between pre- and postoperative means of four-frequency BC and AC that resulted from the two different calculation methods were quite small (< 1.9 dB) compared to the actual value and were therefore not regarded as clinically important. However, the numerical difference when calculating mean postoperative four-frequency ABG using 3 kHz or 4 kHz was large. The proportion of cases reaching ≤ 10 dB and 10–20 dB was also significantly and meaningfully larger when 3 kHz was used instead of 4 kHz. Consequently, our findings show that results cannot be directly compared without taking methods into account. However, while the use of 3 kHz as a reporting frequency might give the surgeon a better outcome, it does not change the patient’s perspective on the outcome. Also, the impact of 3 and 4 kHz respectively on speech discrimination has not yet been investigated and are to our knowledge not known. There is value in including 4 kHz AC measurements in postoperative evaluation of the hearing outcome, since high-frequency hearing loss may be a result of inner ear damage caused by surgery. We urge researchers with access to otosclerosis databases including measured 3 kHz and 4 kHz to investigate whether our findings can be reproduced.

## Conclusion

The success rate in terms of ABG closure differed significantly depending on which of the two calculation methods was used. This was due to a large and disproportionate impact of mean postoperative ABG at 4 kHz on postoperative mean four-frequency ABG, which could have been the result of an anomalous and too low BC measure at 4 kHz. This phenomenon may hamper the interpretation and comparison of surgical results, as well as making surgical results appear worse than they are when using 4 kHz instead of 3 kHz. Substituting 3 kHz with the average of 2 and 4 kHz resulted in a minor but not clinically relevant change of mean ABG. We advocate the inclusion of measured 3 kHz when calculating four-frequency ABG, which is in accordance with the 1995 guidelines from the AAO-HNS, Committee on Hearing and Equilibrium and will enable equal comparisons of the surgical results of otosclerosis surgery.
